# Multiple Dental and Skeletal Abnormalities in an Individual with Filippi Syndrome

**DOI:** 10.1155/2013/845405

**Published:** 2013-10-08

**Authors:** Meera Sandhu, Pooja Malik, Rooposhi Saha

**Affiliations:** Pedodontics and Preventive Dentistry, ITS Center for Dental Studies and Research, C-4, ITS-CDSR, Campus, Muradnagar, Ghaziabad 201206, India

## Abstract

Filippi syndrome is an autosomal recessive condition characterized by variable soft tissue syndactyly of the fingers and toes, microcephaly, pre- and postnatal growth retardation, mildly abnormal craniofacial appearance, and mental retardation. We report on a child with Filippi syndrome who shows syndactyly of fingers, severe postnatal growth retardation, postnatal microcephaly, and moderate to severe mental retardation. In addition, there is a mildly dysmorphic face along with ocular and a number of dental abnormalities. Radiologically, hands demonstrate bony syndactyly, without any hypoplasia of bones. This phenotype can easily be classified in the group of craniodigital syndromes, but it is difficult to make a more clearly defined diagnosis, based on other minor anomalies, because of the presence of overlapping features. On the basis of various pathognomic features, we conclude that our patient could be an additional case of Filippi syndrome. Moreover, newly recognised features in this patient may be due to variability in phenotypic expression.

## 1. Introduction

Filippi syndrome is a rare disorder involving finger and toe abnormalities, a small head, characteristic face, and physical and mental retardation. The number of digits involved and the extent of webbing between digits are variable as are many of the other features. In 1985, Filippi [1] reported a new syndrome with mental retardation, postnatal short stature, unusual faces, syndactyly, and severe microcephaly in two brothers and their younger sister originating from Italy. Here, we report a further observation of Filippi syndrome, with severe skeletal anomalies.

## 2. Case Report

**Figure 1:**
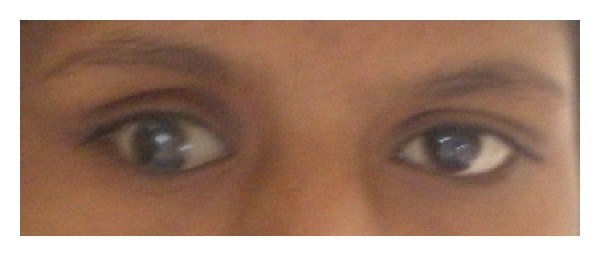
Left eye with an eye prosthesis.

**Figure 2:**
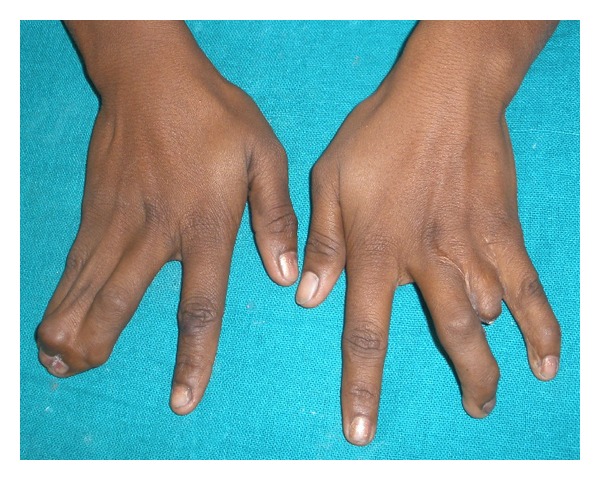
Hands showing syndactyly of fingers.

**Figure 3:**
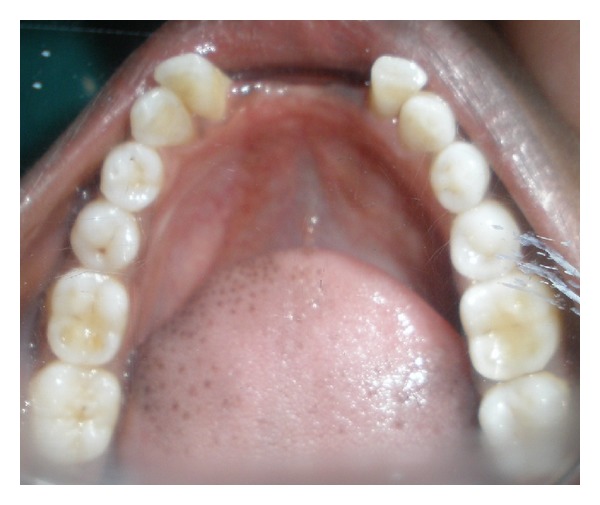
Pretreatment mandibular arch showing missing central incisors.

An eighteen-year-old female reported with the chief complaint of missing teeth in the lower front jaw region. She gave a history of trauma 1 year ago in which she lost her front teeth. No other significant history in terms of medications or family was obtained. She gives a history of a surgical attempt of separating the joined fingers, during which half of her ring finger of the left hand was lost due to postsurgical infection. The prenatal, natal, and post natal history was also nonsignificant. She also had mild to moderate mental retardation. On general examination, she was seen to have an abnormally short stature with a height of 4 feet 6 inches and weight of 30 kgs. She also had a loss of vision in the left eye and was wearing eye prosthesis (Figure 1). She was also seen to have syndactyly in relation to middle, ring, and little fingers of both hands (Figure 2). On intraoral examination, she had good oral hygiene and missing right and left mandibular central incisors (Figure 3).

**Figure 4:**
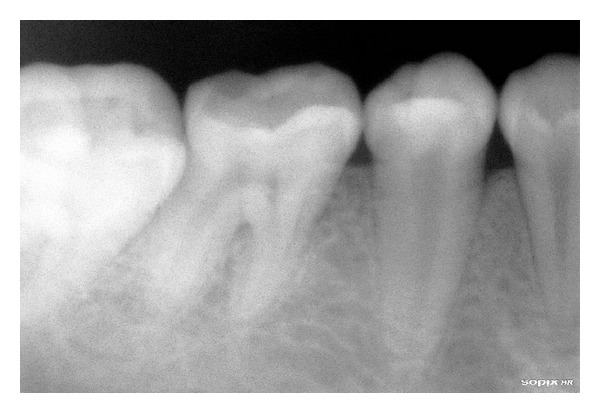
Short crown root length of mandibular I molar and premolar.

**Figure 5:**
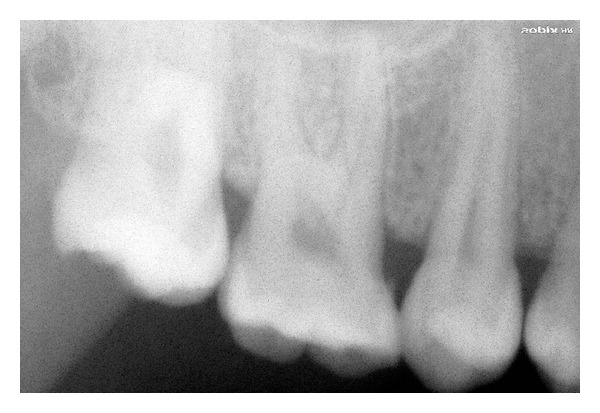
Short crown root length of maxillary I molar.

**Figure 6:**
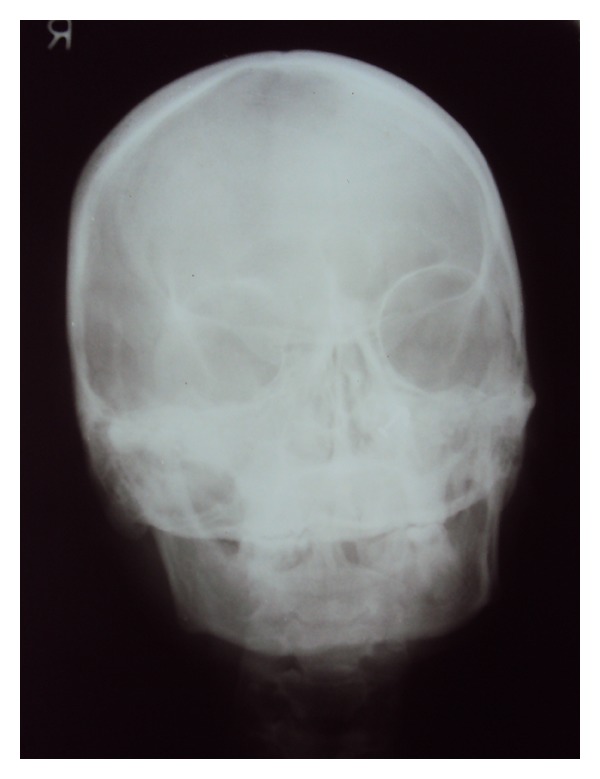
PA view of skull.

**Figure 7:**
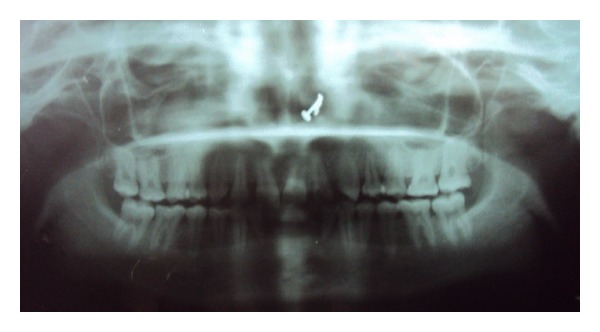
Orthopantomogram.

**Figure 8:**
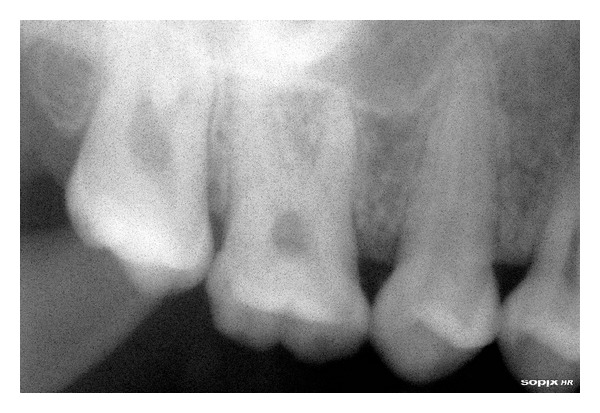
Maxillary second molar with an enlarged pulp chamber.

**Figure 9:**
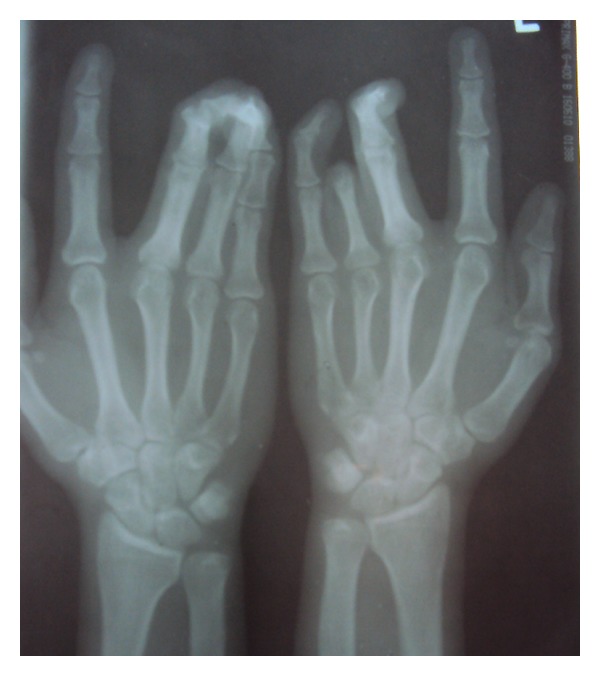
Hand wrist X-ray showing bony union of 3 fingers.

Relative microdontia with respect the crown root length was shorter than normal measurements (as measured on a radiovisuography), by approximately 8 mm, in mandibular right and left first molars and first premolars (Figure 4) and also in maxillary first molars (Figure 5) bilaterally. The soft tissue and other oral mucosal structures did not reveal any abnormality. Various investigations like orthopantomogram, lateral, and posteroanterior (PA) view of skull and hand wrist X-rays were taken to confirm the findings. The PA view (Figure 6) of skull was found to be normal. The orthopantomogram revealed the congenital absence of all the third molars and a generalised horizontal bone loss (Figure 7). In addition to the aforesaid, enlarged pulp chambers with respect to the maxillary right and left second molars (Figure 8) were also observed. Hand wrist X-ray revealed bony union of the middle, ring, and little fingers of both hands (Figure 9).

## 3. Discussion

Filippi syndrome is a very rare disorder involving finger and toe abnormalities, a small head, characteristic face, and physical and mental retardation. The number of digits involved and extent of webbing between digits are variable as are many of the other features. The main characteristics in our patient were post natal growth retardation, bony syndactyly of the fingers, and moderate mental retardation. Other features noted were a number of dental findings like congenitally missing teeth, bone loss inspite of good oral hygiene, short crown root length of certain teeth, and an enlargement of pulp chambers (Taurodontism) (Table 1). In addition, in the present case, a congenital loss of vision has also been reported. These multiple congenital anomalies and mental retardation, (MCA/MR Syndromes) very much resemble these described by Filippi [1] and reported in other families by Meinecke [2]. However, other skeletal anomalies, such as dislocation of the elbows with hypoplasia of the radial heads and speech abnormalities, were not present in our case (Table 2).

**Figure 10:**
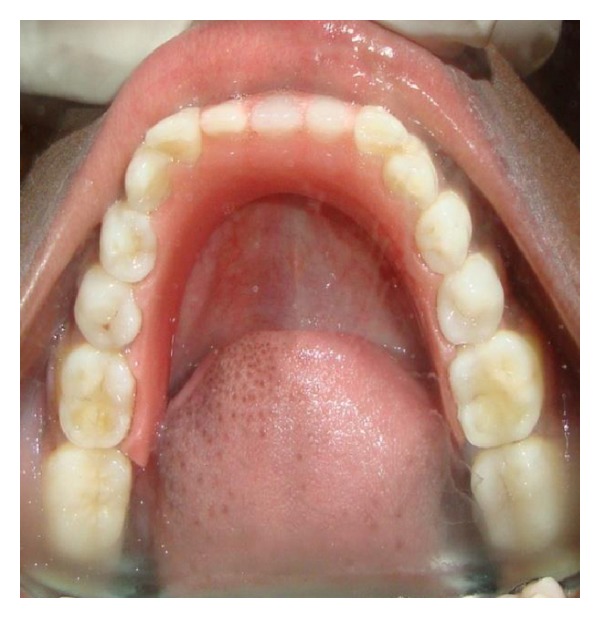
Posttreatment mandibular arch showing removable partial denture.

Occurrence of the disease in both sexes and consanguinity add to the view that an autosomal recessive mode of inheritance is likely in Filippi syndrome [3]. Lorenz et al. [4], Scott et al. [5], and Zerres et al. [6] independently reported two distinct MCA/MR syndromes with microcephaly, syndactyly, and short stature. However, in both cases, the facial appearance is very different from the dysmorphism observed in Filippi syndrome. Thus, Filippi syndrome appears to be an independent and possibly homogeneous genetic condition. In this case, the patient's missing teeth were replaced using a removable partial denture (Figure 10) and recalled for regular follow up checkups.

## Figures and Tables

**Table 1:** Dental findings in this report.

Congenital absence of all third molars
Horizontal bone loss in spite of good oral hygiene
Shortened crown root length in multiple teeth
Enlarged pulp chambers in molars

**Table 2:** Clinical features in six patients with Filippe syndrome.

Sex	Filippi [1]			Meinecke [2]		This report
	M	M	F	M	F	F
Mental retardation	+	+	+	+	+	+
Short stature	+	+	+	+	+	+
Syndactyly (Hands)	+	+	−	+	+	+
Elbow dislocation	−	−	−	−	−	−
Delayed bone age	+	−	?	?	−	−
Dental findings	−	−	−	−	−	+
Visual disturbances	−	−	−	−	−	+
